# Survival and Interplay of γ-Aminobutyric Acid-Producing Psychobiotic Candidates with the Gut Microbiota in a Continuous Model of the Human Colon

**DOI:** 10.3390/biology11091311

**Published:** 2022-09-04

**Authors:** Rojaalsadat Mousavi, Walid Mottawea, Marie-Claude Audet, Riadh Hammami

**Affiliations:** 1 School of Nutrition Sciences, Faculty of Health Sciences, University of Ottawa, Ottawa, ON K1H 8M5, Canada; 2 Department of Microbiology and Immunology, Faculty of Pharmacy, Mansoura University, Mansoura 35516, Egypt; 3 Department of Biochemistry, Microbiology and Immunology, Faculty of Medicine, University of Ottawa, Ottawa, ON K1H 8M5, Canada

**Keywords:** GABA-producing bacteria, probiotic properties, gut microbiota, microbial metabolites, simulated human colon

## Abstract

**Simple Summary:**

Appreciable evidence suggests that gut microbiota interact with the brain and play a key role in the pathogenesis of mental illnesses. Psychobiotics are beneficial bacteria (probiotics) or support for such bacteria (prebiotics) that can positively modulate microbiota–gut–brain interactions. Several trials suggest probiotics are involved in normalizing brain processes related to stress responses and mood improvements. Here, we studied the growth and competitiveness of recently identified GABA-producing psychobiotic candidates in a continuous model of the human colon. In summary, supplementation with these probiotic candidates positively modulated the gut microbiome composition and metabolism, suggesting their suitability for gut health-promoting applications.

**Abstract:**

Over decades, probiotic research has focused on their benefits to gut health. Recently, the gut microbiota has been proven to share bidirectional connections with the brain through the gut–brain axis. Therefore, the manipulation of this axis via probiotics has garnered interest. We have recently isolated and characterized in vitro probiotic candidates producing γ-aminobutyric acid (GABA), a major neuromodulator of the enteric nervous system. This study investigates the growth and competitiveness of selected GABA-producing probiotic candidates (*Bifidobacterium animalis*, *Streptococcus thermophilus*, and *Lactobacillus delbrueckii* subsp. *bulgaricus*) in the presence of human gut microbiota ex vivo in a model mimicking physiological and microbiological conditions of the human proximal colon. Supplementation with GABA-producing probiotic candidates did not affect the overall gut microbiota diversity over 48 h of treatment. However, these candidates modulated the microbiota composition, especially by increasing the *Bacteroidetes* population, a key gut microbe associated with anti-inflammatory activities. The level of microbiota-generated SCFAs within 12 h of treatment was also increased, compared to the control group. Results from this study demonstrate the probiotic potential of the tested GABA-producing bacteria and their impact on gut microbiota structure and metabolism, suggesting their suitability for gut health-promoting applications.

## 1. Introduction

Gut microbiota dysbiosis has been linked to many brain-function and behavioral disorders [1,2]. This link between the gut microbiota and mental disorders had been initially confirmed via animal models, where germ-free (GF) mice developed anti-depression and anti-anxiety phenotypes as compared to specific pathogen-free (SPF) mice, as a result of a hormonal imbalance in the hypothalamic–pituitary–adrenal axis [3,4]. The gut microbiota interacts with the host central nervous system through the gut–brain axis. This interaction could be directly mediated through microbial metabolites, such as neurochemicals or short-chain fatty acids (SCFAs), or indirectly via neuronal, immunological, or endocrinal connections [5]. Therefore, modulation of the gut microbiota may represent a promising alternative biotherapeutic approach to mental disorders.

Probiotics represent the most common way to harness the gut microbiota for a therapeutic benefit. Probiotics are live microorganisms that, when administered in adequate amounts, confer a health benefit to the host [6]. Several beneficial effects have been associated with the consumption of probiotics, such as the improvement of digestion and intestinal transit [7], prevention of food allergies [8], suppression of pro-inflammatory cytokines and upregulation of CD4+ T cells [9], fighting against infectious and antibiotic-associated diarrhea [10], irritable bowel diseases [11], and mental health disorders [12]. Probiotics alter the gut environment by inducing the generation of a myriad of bioactive metabolites, including SCFAs and neurotransmitters [13]. SCFAs, derived from the intestinal microbial fermentation of indigestible fibers by anaerobic microbiota, have been known as a main source of energy for colon epithelial cells, making them crucial to gastrointestinal health and energy metabolism [14].

Several studies have explored the efficiency of probiotics as alternative biotherapeutics in different health conditions. One such application is the use of psychobiotics, which are probiotics able to confer mental health benefits [15]. For instance, two probiotic strains, *Bifidobacterium longum* NCC3001 and *Limosilactobacillus reuteri,* exhibited a neuromodulatory effect by mitigating the action potential of electrically stimulated mesenteric nerves [16,17]. Such modulation of the enteric nervous system mainly arises from the metabolism of neurochemicals like GABA, serotonin, dopamine, or their precursors [5,13]. GABA is an inhibitory neurotransmitter in the enteric and central nervous system that may act on the peripheral nervous system through the gut–brain axis [18]. GABA is synthesized from glutamic acid via the action of the enzyme glutamic acid decarboxylase (GAD) and pyridoxal phosphate (PLP) as a co-factor [19]. Commensal lactic acid-producing bacteria (LAB), including members of the *Bifidobacterium* and *Lactobacillus* genera, were identified to synthesize and deliver GABA [20]. Importantly, GABA has been identified as an essential growth factor that can solely induce the growth of unculturable gut microorganisms [21]. Therefore, this neuroactive metabolite can, in turn, modulate the gut microbiota structure in various kinds of stress [22]. For instance, the relative abundance of *Bacteroides*, a major GABA-producing genus, was negatively correlated with depression-associated brain signatures [21], indicating a significant role of microbiota-derived GABA in brain functionality.

There is a growing interest in identifying the capability of LAB strains to produce GABA; however, the scientific evidence in relation to the capacity of these strains to grow, survive, and produce GABA in vivo, as well as their interaction with the colonic microbiota in physiological colonic conditions, remains rare [23]. The ex vivo screening of these strains for their ability to modulate the gut microbiota composition and functionality and maintain a specific microbial population of interest [24] is thus required before moving promising candidates for in vivo clinical trials. The present study aimed to evaluate the isolated GABA-producing LABs, recently identified and characterized [25], for their capacity as potential GABA-producing probiotic candidates to positively impact gut microbiome composition and metabolism in an ex vivo continuous fermentation model that mimics the physiological conditions of the proximal colon.

## 2. Material and Methods

### 2.1. Bacterial Strains, Media, and Culture Conditions

Bacterial strains were isolated from food cultures described previously [25], and pure overnight cultures of all strains were cultured in Lactobacilli MRS broth (VWR Avantor, Canada) and cryopreserved in 20% glycerol in MRS broth at −80 °C until use. The strains were grown at 37 °C for 24 h anaerobically (Whitley A35 Anaerobic Workstation, UK).

### 2.2. Bacterial Enumeration by Plate Counts

Viable cell counts were determined using the drop plate method. Four 20 μL drops of each 8-fold serial dilution of overnight subculture with peptone water (0.15% *w*/*v*, pH 7.0) were plated in duplicate on selective media for enumerating bacterial colony counts. *Streptococcus thermophilus* and *Lactobacillus delbrueckii* subsp. *bulgaricus* (termed *Lactobacillus bulgaricus* hereafter) were enumerated on M17 and MRS agar after aerobic incubation at 37 °C for 24 h, respectively. *Bifidobacterium animalis* was counted using MRS agar, with 48 h of anaerobic incubation at 37 °C. Viable cell counts were demonstrated as log CFU/mL of the fermentation culture medium.

### 2.3. Human Colonic Fermentation Model

#### 2.3.1. Nutritive Culture Medium

Macfarlane broth is a complex, nutritive medium mimicking the nutrients encountered in a healthy adult large intestine [26]. The nutrient medium was described in [27]. The medium was autoclaved for 15 min, and a filter-sterilized mixture of vitamin solution was added to the cold medium before use.

#### 2.3.2. Fecal Sample Collection and Cell Immobilization in Gel Beads

Fecal samples were obtained from two healthy adult donors (1 male and 1 female), with two distinctive microbiota communities [27], who had not been exposed to antibiotic treatment or probiotic supplements for at least three months before donation. The collection of fecal samples was approved by The University of Ottawa Research Ethics Board and Integrity (ethics file number: H-02-18-347; approval date: 29 July 2019). The feces were processed to slurries by dilution in reduced peptone water (20%, *w*/*v*), homogenized, and further immobilized in 1–2 mm gel beads consisting of gellan gum (2.5%, *w*/*v*), xanthan (0.25%, *w*/*v*), and sodium citrate (0.2%, *w*/*v*) under anaerobic conditions, as described previously in the details [28].

#### 2.3.3. Experimental Setup and Fermentation Procedure

**Figure 1:**
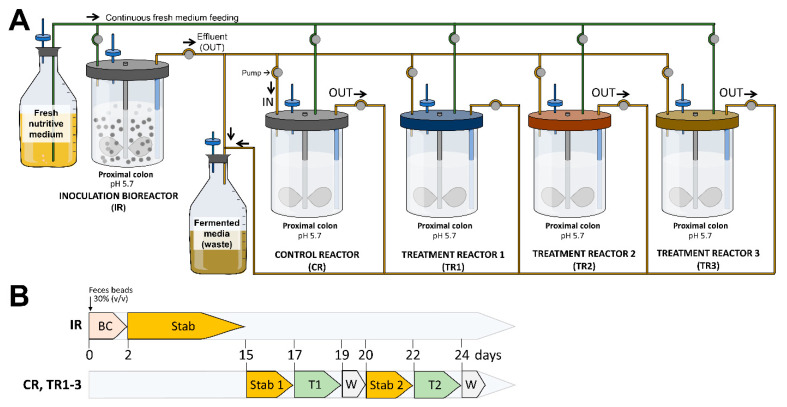
Experimental reactor setup (**A**) and fermentation protocol (**B**) of the ex vivo colonic model. IR: inoculum reactor, containing immobilized donor feces (30% *v*/*v*); CR: control reactor; TR1-TR3: test reactors 1–3; BC: Bead colonization period; Stab: stabilization period; T: treatment period; W: wash period.

The continuous fermentation was carried out using an ex vivo model of the human proximal colon (N*u*GUT Research Platform, University of Ottawa), as previously described [27]. The model consisted of a two-stage design comprising of an inoculation reactor (IR) (1L BioFlo^®^ 120 vessel; Eppendorf, Mississauga, ON, Canada), with immobilized fecal microbiota used to continuously inoculate four second-stage DASGIP^®^ bioreactors (Eppendorf, Mississauga, ON) operated in parallel (Figure 1A). The four subreactors included a control reactor (CR) (no treatment control) and three test reactors (TR1, TR2, TR3). Each reactor was set up to reproduce the physiological and microbiological conditions of the adult proximal colon (pH 5.7, stirring at 120 rpm, 37 °C, and a mean retention time of 8 h). Anaerobiosis was ensured through the continuous headspace flushing of N_2_ and CO_2_ at a 0.9:0.1 ratio, and a constant pH of 5.7 was maintained by adding 2.5 M NaOH. Fermentation was initiated by inoculating the IR containing 140 mL of fresh sterile Macfarlane culture medium with 60 mL of immobilized gel beads (Figure 1B). During the first 48 h, the colonic model was run in a batch mode to favor beads colonization and subsequently switched to continuous mode for the rest of the experiment. After the stabilization of the microbiota by continuous intestinal fermentation for 2 weeks, the four bioreactors were set up and run without any treatment for 48 h to reach the stability of the microbial community. Once the stabilization was reached in all reactors, the bioreactors were subjected to probiotic treatment as follows: CR bioreactor: served as no treatment control; TR1 bioreactor: *S. thermophilus* ST16; TR2 bioreactor: *B. animalis* ST20; and TR3 bioreactor: a mixture of *S. thermophilus* ST16 and *L. bulgaricus* ST7. Probiotic candidates were added once to the corresponding test reactor at a final concentration of 10^9^ CFU/mL each. Collected effluent samples (2 mL) were taken in duplicates and centrifuged at 14,000× *g* for 5 min every 2 h during the first 12 h of treatment (0, 2, 4, 6, 8, and 12 h) and then at 24 and 48 h from the bioreactors. The collected samples were separated (centrifugation at 14,000× *g*, 5 min, 4 °C) into a pellet used for genomic DNA extraction and supernatant for short-chain fatty acid (SCFA) analysis. Each fermentation experiment was conducted in duplicates for each fecal sample donor (a total of 4 biological replicates).

### 2.4. Microbiota Diversity Analyses

#### 2.4.1. Genomic DNA Extraction

The genomic DNA extraction was performed on an up to approximately 100 mg of wet-weight microbial cell fermentation pellet using the FastDNA^®^ Spin Kit (MP Biomedicals; Solon, OH, USA) and homogenized with the Bead Mill-24 homogenizer (Fisher Scientific; Ottawa, ON, Canada), as described by [27]. The initial DNA concentration (200 ng/μL) was quantified by the Qubit Fluorometer using the Qubit dsDNA BR Assay Kit (Qubit™ Flex Fluorometer, ThermoFisher Scientific, Wilmington, NC, USA).

#### 2.4.2. High-Throughput 16S DNA Sequencing

The microbial composition and diversity of the fecal and effluent fermentation samples were assessed using high-throughput sequencing of the 16S rRNA gene on the platform MiSeq (Illumina, CA, USA). The V3–V4 regions of the 16S rRNA gene were amplified using dual-barcoded primers, and the amplicon library for sequencing was constructed using the Illumina standard protocol. The amplicon libraries were pooled in equimolar amounts and paired-end sequenced with Illumina MiSeq platform (N*u*GUT Research Platform, University of Ottawa) using the 600 bp MiSeq Reagent Kit v3 (Illumina; San Diego, CA, USA), as per standard protocol. Raw sequences were demultiplexed, and adapters were truncated. Sequences were quality filtered based on a minimum quality score of 20, denoised using the default parameters of the deblur plugin wrapped by QIIME 2.2020.8, and had a minimum paired read length of 439 nucleotides. Afterwards, the high-quality sequences were clustered into observed features based on 97% similarity using the Greengenes database (v13.8) via the QIIME 2.2020.8 software [29]. The observed features were rarefied into an equal number of 4000 reads per sample using QIIME before conducting the diversity analyses.

#### 2.4.3. qPCR Analysis

Quantitative PCR (qPCR) was performed, as described by [30], on a Bio-rad CFX96 Real-Time PCR detection system (Biorad, Oakville, ON, Canada) in 96-well plates. Specific primers used in this study are summarized in Table 1. The qPCR reaction mixture (20 μL) contained 1× SsoAdvanced Universal SYBR Green Supermix (Bio-Rad; Mississauga, ON, Canada), 0.5μM of each forward and reverse primer (Millipore-Sigma, Cleveland, OH, United States), 6μL of DNase-free water (Invitrogen), and 25 ng of extracted DNA. qPCR quantification of each sample was performed in duplicates. The amplification program was set up for 98 °C for 3 min, followed by 40 cycles of 95 °C for 15 s and 60 °C for 30 s, and then followed by a melting cycle of the PCR product from 65 °C to 95 °C. Cq values were extracted using the Bio-Rad CFX Maestro software, and the relative abundance of each organism was determined as a ΔCq value (taxon Cq–universal 16S rRNA Cq), as described before [27], where the increase in ΔCq indicates a decrease in the relative abundance, and the opposite is also true.

### 2.5. Determination of Production of SCFAs Using GC

The production of SCFAs butyrate, acetate, and propionate in fermentation samples from all sub-reactors was determined using gas chromatography coupled with the flame ionization detector (GC-FID) (Shimadzu GC-2030), as previously described [34]. In brief, 2 mL of supernatants of collected effluent-fermented samples were centrifuged twice at 14,000× *g* for 30 min at 4 °C and filtered with a 0.22 μm membrane. 2-ethyl butyric acid was used as an internal standard and added to each sample at a concentration of 0.5 mM. The analysis was carried out using a capillary column Stabilwax-DA (60 m × 0.25 µm; Restek), at a flow rate of 0.4 mL/min, with 2 μL injected into the GC (Nexis GC-2030, Shimadzu, Japan). Helium was used as the carrier gas on the flame ionization detector (FID). The initial temperature of the oven was 100°C and then was increased to 200 °C at a rate of 10 °C/min. Injector and detector temperatures were maintained at 200 °C and 300 °C, respectively. The peaks were identified by comparing their retention times with the volatile acid standard mix from Millipore Sigma (Oakville, ON, Canada). The data collection was analyzed using Lab Solutions software developed by Shimadzu Corporation, Japan. All samples were analyzed in duplicates, and results were expressed in mM.

### 2.6. Statistical Analyses

Alpha diversity was estimated with observed features, Shannon entropy, Pielou’s evenness, and Faith_PD. Beta diversity among samples was calculated using the Bray–Curtis distance and visualized using principal coordinate analysis (PCoA). The contribution of different treatments to the diversity of the gut microbiota community was assessed from the Bray–Curtis distance matrix using the permutational multivariate analysis of variance (PERMANOVA) pairwise and 999 permutations [35]. To identify differential taxa among different treatments, a linear discriminant effect size analysis was conducted on the relative abundance of different taxa levels [36]. Samples were labeled, with the treatment type as the sample class and the time points as the subclass. Taxa with a log_10_ LDA score ≥ 2 and a *p* < 0.05 were considered significant. When required, the Kruskal–Wallis test was applied for statistical analysis, and *p*-values were corrected using the two-stage Benjamini, Krieger, and Yekutieli false discovery rate (FDR) procedure. For SCFA contents, statistical analysis was performed using GraphPad Prism software (GraphPad 8). Statistical comparisons were conducted among different treatments at the same time and among different time points within each treatment, using repeated measures two-way ANOVA, with Tukey’s multiple comparisons test and Dunnett’s multiple comparisons test for SCFAs and qPCR results analyses, respectively. Significant differences were indicated in the figures by different *p* values.

## 3. Results

### 3.1. Gut Microbiota Diversity

**Figure 2:**
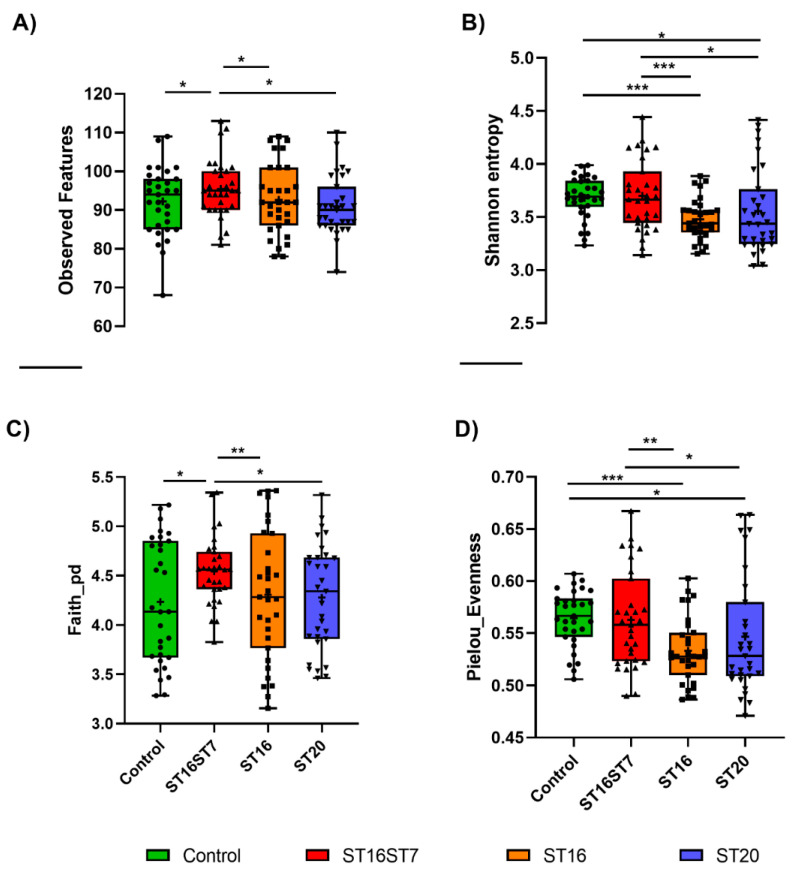
Observed features (**A**), Shannon indices (**B**), Faith’s_PD (**C**), and Pielou’s evenness (**D**) estimates of the identified microbiota in each sample, from bioreactors with either a no-treatment control, or treated with ST16-*S. thermophilus*, a combination of ST7-*L. bulgaricus* and ST16-*S. thermophilus,* or ST20-*B. animalis*. Data were analyzed using the Kruskal–Wallis test and a ywo-stage Benjamini, Krieger, and Yekutieli FDR procedure (* *p* < 0.05, ** *p* < 0.01, *** *p* < 0.001).

Four diversity indices were calculated to compare the alpha diversity of different treatments; observed features, Shannon entropy, Faith_PD, and Pielou’s evenness. Dual treatment with the combination of *L. bulgaricus* and *S. thermophilus* (ST16ST7) strains exhibited a significant increase in microbiota diversity, as indicated by the increase in the number of observed features and Faith_PD, in comparison to the no-treatment control group, and the increase in different indices, as compared to other treatments (Figure 2, *p* < 0.05).

**Figure 3:**
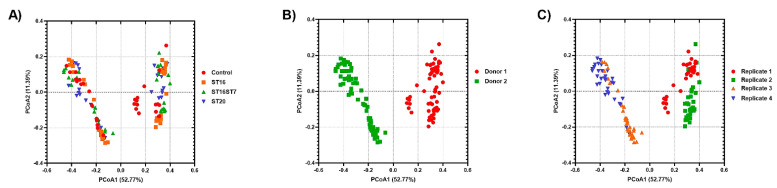
Plots of principal coordinate analysis (PCoA) based on Bray–Curtis distances among the identified microbiota in different samples, showing clustering based on treatment (**A**); donor (**B**), and replicate (**C**). The samples were colored as indicated in the legends. PCoA1 and PCoA2 represent the top two coordinates that captured the highest microbial variability among samples, and the percentage shown indicates the fraction of variation represented by each coordinate. Permutational multivariate analysis of variance (PERMANOVA) was used to test for the statistical significance of sample grouping.

Beta diversity was calculated to identify which factor controlled the microbiota diversity among different samples using Bray–Curtis distances and visualized using principal coordinate analysis (PCoA) across treatments, donors, and biological replicates. Plots of PCoA are shown in Figure 3. The microbiota of different treatments were clustered by the donor (PERMANOVA = 153.177, *p* = 0.001; 999 permutations), by the biological replicate within each donor (PERMANOVA = 3.53, *p* = 0.003; 999 permutations), and by the treatment within each experiment replicate (PERMANOVA = 2.45, *p* = 0.003; 999 permutations).

### 3.2. Effect of GABA-Producing Probiotic Candidates on Gut Microbiota Composition

**Figure 4:**
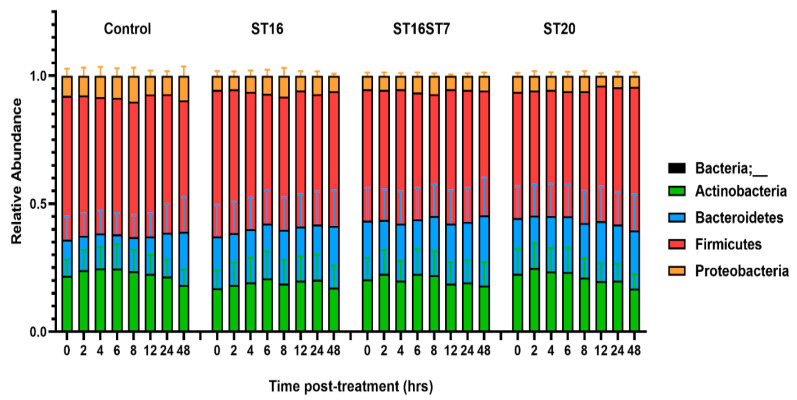
The composition of gut microbiota treated with GABA-active strains over 48 h in the reactor setup of the human colonic ex vivo model. Four bacteria phyla were identified in the generated dataset, including Firmicutes, Bacteroidetes, Proteobacteria, and Actinobacteria.

We determined the change in gut microbiota composition over 48 h post treatment for *B.* animalis ST20, *S. thermophilus* ST16, and the combination of *L. bulgaricus* ST7 and *S. thermophilus* ST16, as compared to the no-treatment control. The developed microbiota in different sub-reactors were generally composed of the four major phyla Firmicutes, Bacteroidetes, Actinobacteria, and Proteobacteria (Figure 4).

**Figure 5:**
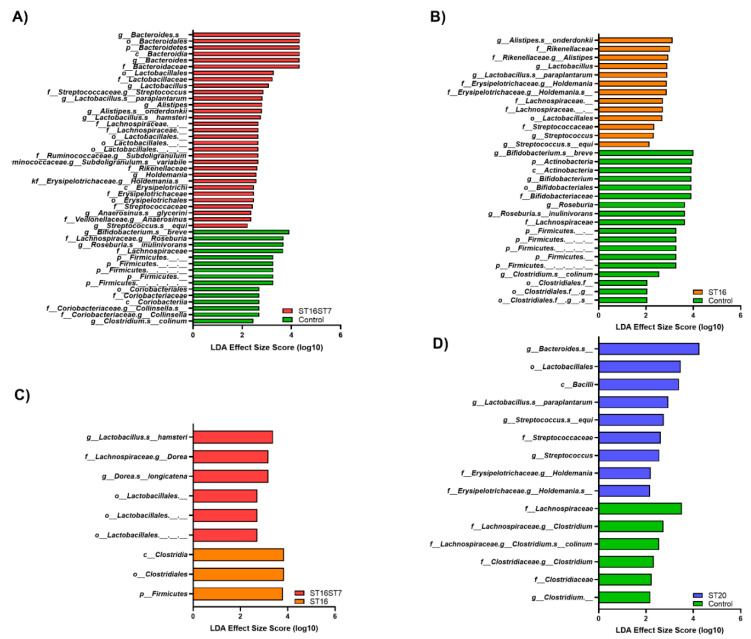
The test probiotic candidates modulating gut microbiota composition. (**A**–**D**) Histograms of the linear discriminant analysis (LDA) scores showing microbial taxa that vary significantly in abundance between: (**A**) no-treatment control and test treatment of the combination of *L. bulgaricus* and *S. thermophilus*, (**B**) no-treatment control and test treatment of *S. thermophilus,* (**C**) compare test treatment of *S. thermophilus* and combination of *L. bulgaricus* and *S. thermophilus*, (**D**) no-treatment control and test treatment of *B. animalis*.

Linear discriminant analysis (LDA) demonstrated the microbial taxa at different phylogenetic levels affected by the treatments, as shown in Figure 5. The microbiota composition subjected to the combination of *L*. *bulgaricus* ST7 and *S. thermophilus* ST16 treatment was enriched in *Bacteroides* and *Lactobacillus* at the genus level; *Streptococaceae*, *Ruminococcaceae*, *Lactobacillaceae,* and *Bacteroidaceae* at the family level; and the Lactobacillales order, compared to untreated microbiota (Figure 5A; *p* < 0.05). The microbiota treated with *S. thermophilus* ST16 exhibited an increase in *Lactobacillus*, *Alistipes,* and *Streptococcus* at the genus level; *Rikenellaceae* and *Erysipelotrichaceae* at the family level; and Lactobacillales at the order level, as compared to untreated microbiota (Figure 5B; *p* < 0.05). In contrast, *S. thermophilus* ST16 treatment led to a major modification of microbiota with enrichment of the Firmicutes phylum and the Clostridiales order, compared to the combination of *L*. *bulgaricus* ST7 and *S. thermophilus* ST16 treatment, which did not affect the microbiota at the phyla level, but depleted *Clostridiaceae* at the family level (Figure 5C; *p* < 0.05). Microbiota subjected to the *B. animalis* ST20 treatment was also enriched in *Streptococaceae* and *Erysipelotrichaceae* at the family level; in *Bacteroides, Lactobacillus*, and *Streptococcus* at the genus level; and in *Lactobacillales* at the order level, compared to the control microbiota (Figure 5D; *p* < 0.05).

### 3.3. Microbial Survival Analysis by qPCR

**Figure 6:**
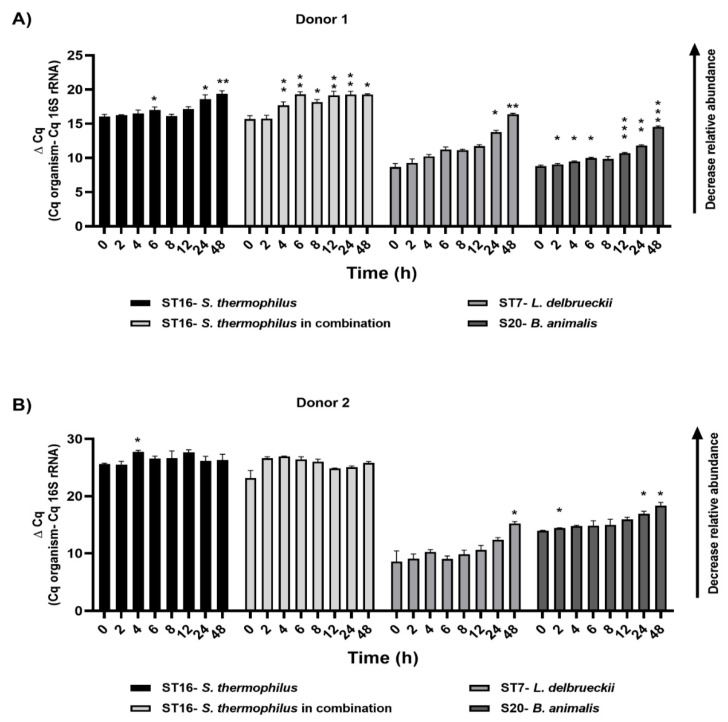
Relative abundance of target species identified by qPCR, as indicated by ΔCq: relative abundance of inoculated probiotic candidates in various bioreactors with donor 1 (**A**) and donor 2 (**B**) microbiota over 48 h post treatment. The increase in ΔCq indicates a decrease in the relative abundance. Repeated measures ANOVA with Dunnett’s multiple comparisons test was used for statistics. Significant differences were indicated for each time point compared to the zero time (* *p* < 0.05, ** *p* < 0.01, *** *p* < 0.001).

We monitored the levels of the probiotic candidates in their corresponding bioreactors via quantitative PCR and by using primers specific to the added strains. We were able to detect all the strains after 48 h of treatment in their corresponding sub-reactor. The relative abundance of all the strains decreased gradually over the 48 h and reached a significant reduction in their levels after 24 h and 48 h of the treatment in donor 1. For donor 2, we noticed the same reduction trend in their relative abundance over time; however, no significant difference was detected statistically (Figure 6).

### 3.4. Effect of GABA-Producing Probiotic Candidates on Microbiota Generation of SCFAs

**Figure 7:**
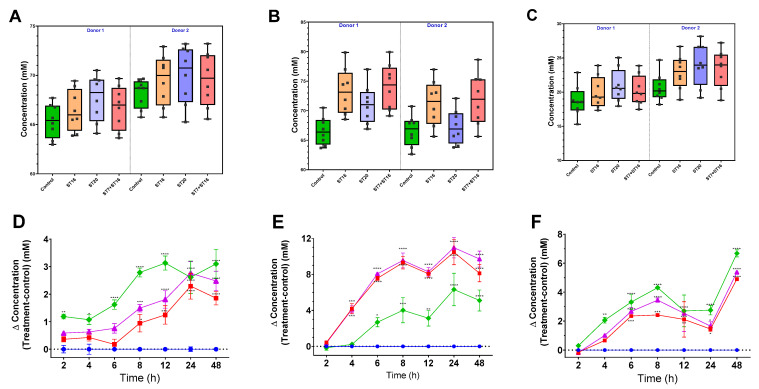
Concentration (panels (**A**–**C**); separated by donor) and concentration fold increase over control (panels (**D**–**F**); combined datasets) (Δ concentration = test-treatment−control) of short-chain fatty acids (SCFAs) measured by GC over 48 h: butyrate (**A**,**D**), acetate (**B**,**E**), and propionate (**C**,**F**) from top to bottom for each time point, with two biological replicates for donors 1 and 2 (**A**–**C**) over 48 h. Control (circle, blue); treatment with ST20 (diamond, green); treatment with ST16 (square, red); and treatment ST16 in combination with ST7 (triangle, purple). Statistical comparisons were conducted using repeated measures 2-way ANOVA test with Dunnett’s multiple comparisons test. Significant differences were indicated for each time point compared to the control time (* *p* < 0.05, ** *p* < 0.01, *** *p* < 0.001, **** *p* < 0.0001).

We quantified the generation of SCFAs by the developed microbiota in response to the candidate probiotic treatments using GC. We detected the three major SCFAs known to be generated by the gut microbiota, including acetate, butyrate, and propionate. Inoculation of GABA-producing bacteria induced a significant increase in the three metabolites in both donors (*p*< 0.05; Figure 7). For instance, treatment with *B. animalis* ST20 induced the most significant increase in butyrate and propionate, compared to the control group, but had a less pronounced effect on acetate production than treatment with ST16 alone or in combination with ST7 (Figure 7).

## 4. Discussion

This study explored three active GABA-producing LAB strains as potential psychobiotic candidates by assessing their interplay with the gut microbiota and their impact on the microbiota’s structural and functional profiles. We employed an ex vivo model, which reproduced a stable microbial ensemble mimicking the human colon microbiota. The identified microbiome features belonged to the four major phyla, Firmicutes, Bacteroidetes, Actinobacteria, and Proteobacteria, known as common phyla of the gut microbiota, with a predominance of Bacteroidetes and Firmicutes [37]. We developed two distinct communities from healthy donors. Both microbiota communities were distinctively different at the family level, reflecting the stool microbiome of the two donors, dominated by Bacteroidaceae, Lachnospiraceae, and Veillonellaceae for the first donor, and enrichment of Lachnospiraceae and Veillonellaceae, with low abundance of Bacteroidaceae, for the second donor, as presented before by our group [27]. However, some variations between the donor stool microbiota and the one developed in the bioreactor were expected, as a result of the variability of the microbiota at different anatomical locations of the gut, including the variabilities between the proximal colon adopted in our system and the donor stool microbiota [38]. Still, we have to consider some limitations of the ex vivo colon simulator models, such as a low number of biological replicates, which is due to the time and the high-cost constraints, short period of microbial treatment, enrichment/depletion effect of the culture medium, and the lack of complexity of the gut mucosal environment [27,39].

Mental health disorders have been associated with depleted gut microbiota diversity [40,41]. For instance, there is an inverse correlation between gut microbiota diversity and the clock gene (ARNTL gene) methylation in bipolar disorder patients [42]. Hence, we assessed the influence of the three psychobiotic candidates, isolated in a previous study [25] on microbiota diversity, in a simulated human colon. Interestingly, the combination of *L. bulgaricus* and *S. thermophilus* increased microbiota diversity over 48 h of treatment. Recently, dietary GABA supplementation has been reported to increase gut microbiota diversity in an *E. coli*-infected piglet model [43]. In addition, the administration of *Lactobacillus* cocktails has been reported to increase microbiota diversity post-antibiotic treatment [44,45]. Additionally, it has been reported that LPS-induced decreases in gut microbiota diversity in a mouse model was restored by a single strain of *S. thermophilus* [46]. Combining two *Lactobacillus* strains and *S. thermophilus* also improved the microbiota diversity in neonatal piglets [47]. However, treating the microbiota with a single GABA-producing strain did not alter microbiota diversity, suggesting that the short time of the treatment possibly limited the capacity of these strains to alter the microbiota diversity.

In addition to enhancing microbiota diversity, treating the gut microbiota with the three GABA-generating strains led to significant changes in the microbiota structure. An increased abundance was observed in the genera *Lactobacillus* and *Streptococcus*, which was expected and may be attributed to the test treatment that contained *S. thermophilus* and *L. bulgaricus.* Hence, administering a single dose of these strains maintained a stable level of *Lactobacillaceae* and *Streptococcaceae* families over at least 48 h of treatment. This outcome was also confirmed by the qPCR results. Our data revealed that *Bacteroides*, as one of the major taxa identified in the generated dataset, was increased by GABA-producer treatments. In agreement with that, a previous administration of a mixture of four *Lactobacillus* strains isolated from fermented food increased the levels of mice Bacteroidetes depleted by antibiotic consumption [44]. Analyses of human fecal samples illustrated that *Bacteroides spp*. may produce large quantities of GABA [21]. Additionally, the relative abundance levels of fecal *Bacteroides* have been negatively associated with depression [21]. Moreover, we observed enrichment of *the Lactobacillaceae family after adding* GABA-producing bacteria. Enhancement of *Lactobacillus* has been associated with decreasing depression symptoms in patients with major depressive disorder (MDD), reviewed in [13]. Similarly, the *Erysipelotrichaceae* family was increased after GABA-producing bacteria treatment. The role of the *Erysipelotrichaceae* family in health benefits is unclear. Some studies have reported a correlation between the abundance of *Erysipelotrichaceae* and disease phenotypes, such as inflammatory bowel disease [48,49]. In addition, the microbiota treated with GABA producers were significantly depleted in Clostridiales and *Lachnospiraceae*, while the microbiota treated with the combination of *L. bulgaricus* and *S. thermophilus* strains enriched the *Ruminococcaceae* family, which has been known to have a beneficial effect on gut barrier functions and also introduced as adjuvants to immune checkpoint inhibitors [50]. While the microbiota of individuals with depression is characterized by a disturbed abundance of *Bacteroidaceae* members, bipolar depression is associated with dysbiosis of *Lachnospiracea-* and *Ruminococcaceae*-related taxa [51]. So far, the lack of consistent, distinct microbial signatures for specific mental disorders warrants the hypothetical applications of psychobiotics as personalized adjunct treatments, according to the disease subtypes and the microbiota baseline structure.

SCFAs are saturated fatty acids with mainly acetate, butyrate, and propionate are distributed in the intestine [52] and have been shown to alleviate psychological stress-associated alterations in behaviors, respond to stressors and intestinal permeability, and exhibit antidepressant and anxiolytic effects [14]. Most gut-generated SCFAs are absorbed into the circulation, with a minor part secreted in the feces [52]. We detected high amounts of acetate and butyrate in our colon model; however, propionate concentration in the effluent samples was low. Usually, the ratio of acetate:propionate:butyrate is 60:20:20 [52,53], which was not the case in our results. This may be attributed to the high amounts of indigestible fibers and the predominance of the phylum Firmicutes in our datasets, compared to Bacteroidetes. However, the same base level was detected between different bioreactors before starting the intervention. The three tested probiotic formulas induced a significant increase in the production of SCFAs, especially butyrate, which concur with the observed increase of the butyrate-producing taxa. SCFAs have an essential role in intestinal barrier integrity and immune-modulatory properties [54], implying the potential use of these strains as adjuvant biotherapeutics, not only in mental health disorders, but also in many other conditions characterized by the depletion of SCFAs, such as inflammatory bowel diseases [37,53], irritable bowel syndrome [52], and colorectal cancer [55]. Additionally, they could mitigate the mental symptoms associated with these disorders. For instance, a previous randomized clinical trial has shown that butyrate-producers, including *Lactobacillus,* could decrease psychological symptoms associated with irritable bowel syndrome, such as depression and anxiety [56].

## 5. Conclusions

We herein illustrate that GABA-generating probiotics have the capability to modulate the colon microbiota and enhance the production of SCFAs in a part of the gut. This work also provides evidence that such potential probiotics could be further exploited as functional food products and biotherapeutic regimens to mitigate human health disorders associated with gut microbiota dysbiosis, including mental health disorders. Further research is still required to investigate the survival of GABA-producing bacteria in various gut parts and their ability to produce GABA as an inhibitory neurotransmitter in vivo after oral consumption. We only tested the effects of three food-isolated probiotics belonging to the *Bifidobacterium*, *Lactobacillus*, and *Streptococcus* genera. Future work may investigate other human-originated, GABA-generating gut bacteria and their impact on gut microbiota and disease status.

## Figures and Tables

**Table 1:** Specific primers used for the qPCR.

No	Specificity	Primer Name	Primer Type	Sequence (5′-3′)	GC Content (%)	Melting Temp (°C)	**Ref.**
1	*Lactobacillus delbrueckii* subsp. *bulgaricus*	LdelbF	Forward	GGRTGATTTGTTGGACGCTAG	47.6	66.9	[31]
		LdelbR	Reverse	GCCGCCTTTCAAACTTGAATC	47.6	66. 7	
2	*Streptococcus thermophilus*	S. thermophilusF	Forward	TTATTTGAAAGGGGCAATTGCT	36.3	65.2	[32]
		S. thermophilusR	Reverse	GTGAACTTTCCACTCTCACAC	47.6	58.8	
3	*Bifidobacterium animalis*	IDB61F	Forward	GCATGTTGCCAGCGGGTGA	63.1	73.1	[33]
		IDBC1R	Reverse	ATCCGAACTGAGACCGGTT	52.6	63.5	

## Data Availability

The generated 16S-rRNA sequences were deposited in the NCBI Sequence Read Archive under PRJNA759620.
